# Exploring the effectiveness and awareness of Anki flashcard application on medical student’s academic performance: a cross-sectional study from Saudi Arabia

**DOI:** 10.3389/fmed.2026.1896043

**Published:** 2026-07-31

**Authors:** Osama Alnaser-Almusa, Muhammad Raihan Sajid, Abduljalil El-Sibai, Osama Ammar, Sarah Aldahoul, Tarek El Hayek, Hala Tamim

**Affiliations:** 1 College of Medicine, Alfaisal University, Riyadh, Saudi Arabia; 2 Department of Kinesiology and Health Science, York University, Toronto, ON, Canada

**Keywords:** AFC, digital flash cards, medical education, medical students, Saudi Arabia, spaced retrieval

## Abstract

**Background:**

AFC flashcards (AFC) is a widely used digital flashcard application that allows users to create personalized flashcards, track progress, and receive feedback. A growing number of medical students at Alfaisal University have integrated AFC into their study routines. This study explores how medical students utilize AFC to learn and retain medical knowledge, and whether it helps grasp both pre-clinical and clinical concepts.

**Methods:**

A cross-sectional study was conducted using a validated self-administered questionnaire distributed to medical students from 1st to 6th year at Alfaisal University. A representative sample of 302 participants was calculated from an estimated population of 1400 medical students (95% confidence level, 5% margin of error). The questionnaire assessed AFC awareness, frequency of use, preferred features, and self-reported academic performance. Ethical approval was obtained (IRB No. 20248), and anonymity was ensured.

**Results:**

Of 306 surveyed students, students used AFC more frequently for memorization-based and basic science subjects. The primary self-reported benefit was information retention (89.1%), while the main barriers were time commitment (73.7%) and software unfamiliarity. Although a majority of AFC users perceived a positive association between AFC use and academic performance, multiple linear regression analysis revealed no statistically significant association between AFC use and CGPA (β = 0.04, 95% CI: −0.08 to 0.16, *p* = 0.52). These findings should be interpreted with caution given the cross-sectional design and reliance on self-reported data.

**Conclusions:**

This study demonstrates the widespread use of AFC among medical students at Alfaisal University and its perceived benefits for learning and retention. While a significant correlation between AFC use and higher CGPA was not established, AFC may serve as a valuable supplementary tool for medical students, particularly for memorization-based subjects. Further research is needed to explore optimal AFC use and its impact on academic outcomes in broader medical student populations.

## Background

Amid the rapidly growing body of medical information, medical students are required to master large amounts of information at a rapid pace. Current projections indicate that medical information doubles every 73 days, increasing the demand on students to retain information within the same exam preparation timeframe (1). Consequently, students have turned to third-party platforms to aid their learning process. However, with the wide diversity of available resources, students must prioritize which platforms maximize their time efficiency. Notably, a digital flashcard application known as Anki Flashcards (AFC) has gained significant attention in recent years (2).

AFC utilizes spaced repetition, a technique where information review intervals increase to optimize memory retention and has become increasingly popular among medical students since its creation in 2006 (3). This trend is supported by a study conducted by Deng et al. which showed that students who used AFC with 1,700 flashcards improved their licensing exam scores by one point, though the practical educational significance of a single-point gain warrants careful interpretation (2). While numerous third-party resources are designed for the USMLE Step 1 examination, which assesses foundational basic science knowledge including anatomy, biochemistry, physiology, pathology, pharmacology, and microbiology (4), data on their integration within medical curriculums and impact on student performance remains scarce in the literature (5). Chiang et al. reported the primary benefit was information retention (89.1%), while the main barriers were time commitment (73.7%) and software unfamiliarity. Although a majority of AFC users perceived a positive association between AFC use and academic performance, no statistically significant correlation was found between AFC use and CGPA. These findings should be interpreted with caution given the cross-sectional design and reliance on self-reported data (5). However, the growing popularity of game-based study tools in medical education, as evidenced by a study showing a 50% increase in usage between 2018 and 2021, suggests a similar trend for AFC (6). This implies that AFC use by medical students might be on the rise, though its effectiveness within curriculums requires further investigation.

AFC has not only experienced a surge in usage (7), but it has also become a prevalent method for studying medical content across various institutions. One study revealed that one-fourth of respondents reported that AFC was ineffective in retaining basic science material, while the remaining three-fourths reported that AFC was helpful in doing so (5). Furthermore, 84% of participating students had used AFC at some point in their academic careers, with 139 reporting using it for at least one semester (8).

A review of 120 papers on spaced learning in medical education revealed that spaced repetition platforms, such as the flashcard application AFC, have become ubiquitous among medical students preparing for classroom and board examinations (8). Studies have shown that students who implement these programs into their study routines score higher on standardized licensing examinations (8).

One study found that AFC users had higher USMLE Step 1 scores, and consistent use was correlated with better performance on the exam (7). Another study also had a significant positive correlation between daily AFC use and increased Step 1 score. Possible explanations for the increased Step 1 score include increased efficiency, lower stress, or more solidified routines leading to improved sleep. However, there was no significant difference between AFC and non-AFC users for Step 2 scores (9). The authors speculated that this may be because Step 2 CK places greater emphasis on clinical reasoning and synthesis of information rather than pure factual recall, which may be less effectively supported by flashcard-based memorization (9).

Student’s value AFC’s presence in medical curricula (10), despite the fact that it might not be as effective in improving exam outcomes as it is in improving USMLE step 1 scores. One study revealed that flashcards are the preferred study tool used by students to acquire visual skills for identifying histological images and could be incorporated when designing online study tools. The study suggested that digital flashcards can be a useful tool to support the visual skills of medical students in histology education (6). Another study that assessed the usage and satisfaction of medical students with electronic flashcards based on a psychiatry curriculum, provided first-year medical students with a set of pre-made flashcards to use during the course. The majority (87%) of flashcard users found them helpful and would recommend them to others, however, the usage of flashcards was not associated with higher exam scores (10).

AFC’s popularity is not limited to medical schools alone, as it has also gained a strong foothold in residency programs. Medical practitioners have acknowledged and appreciated its utility in enhancing learning and knowledge retention. For instance, one study examined the use of spaced repetition learning (SRL) through AFC flashcards for improving basic science knowledge among orthopedic surgical trainees (11). The results showed a positive correlation between time spent using the flashcard app and exam scores. Participants who scored the highest reviewed the entire question pool, suggesting that exposure to the full material is crucial for high exam scores.

Similarly, another study evaluated the usage, satisfaction, and effectiveness of a novel electronic flashcard self-study resource on the AFC platform for OB-GYN residents preparing for the 2019 CREOG exam. All flashcard users found them helpful and would recommend the resource to other residents (12, 13).

In one study some students expressed the opinion that the sheer volume of some AFC decks felt overwhelming and that some decks included information outside the scope of the syllabus, in addition to requests for more visual schematics (13). Furthermore, another study aimed to compare the effectiveness of repetitive testing and studying in learning key factual knowledge in clinical nephrology. The study found that repetitive testing was more effective in promoting better recall after 1 week, but no difference was observed after 6 months. The study also suggests that students may not be aware of the superior short-term effectiveness of repetitive testing in supporting knowledge retention. This finding is important because it suggests that educators should consider incorporating more repetitive testing into their teaching methods to help students retain information in the short term (14).

A different study explored the impact of flashcards on first-year medical students (MS1s) in their Medical Gross Anatomy course. It compared the effects of AFC flashcards created by second-year students (MS2s) with third-party flashcards or no flashcards at all. Students who used third-party AFC cards reported having more test anxiety than students who did not, due to the lack of alignment with the specific medical school’s curriculum. However, there was no significant difference in test anxiety between MS1s using MS2-made AFC cards and those who did not (15).

One article conducted a literature review to identify the best practices of expanded-retrieval platforms. These practices included categorical information, schema formation, dual coding, concrete examples, elaboration, change in text, and interleaving. It was found that such practices can help mitigate retrieval-induced forgetting (RIF) by strengthening the memory of presented information, reducing the memory of absent information, and by allowing for the viewing of material not explicitly tested but present upon presentation, such as supporting information on flashcards. Additionally, it was found that multiple choice has been shown to have a lack of benefit, as answer choices do not facilitate proper comparison of information (16).

In another article, many tips on how to establish and maintain a collaborative digital flashcard project based on the undergraduate medical curriculum were discussed. Some of the most important tips shared were the inclusion of collaboration, establishing roles and responsibilities, and having a shared system for organizing and monitoring workload. It was also noted that digital flashcards are easier to share, create, and transport than traditional paper ones. The authors recommended to frame content carefully and to format digital flashcards in a way that improves the clarity of concepts. The flashcard project can serve as a lifelong study tool for practicing medicine, with high-yield clinical content flashcards identified and studied after pre-clerkship (17).

Despite evidence of AFC’s effectiveness for standardized medical exams like the USMLE, a gap exists in the literature regarding its integration within medical school curriculums and its impact on student GPAs in non-US settings. In the present study, we aimed to address this specific gap by exploring AFC awareness and its effectiveness at Alfaisal University’s college of medicine, with particular attention to how AFC usage correlates with students’ perceptions and academic performance (CGPA) in a non-US medical education context. Furthermore, the study intends to contribute to the existing body of knowledge for a specific medical school in the GCC region by investigating AFC usage patterns and effectiveness among medical students at Alfaisal University.

### Theoretical framework

The present study is conceptually grounded in two complementary theoretical frameworks: Spaced Repetition Theory and Cognitive Load Theory.

Spaced Repetition Theory, rooted in Ebbinghaus’s forgetting curve, posits that information is more effectively retained when reviewed at progressively increasing intervals (18, 19). This principle, which forms the algorithmic foundation of AFC, leverages the spacing effect—the phenomenon whereby distributed learning sessions enhance long-term memory consolidation compared to cramming (20). The theoretical premise is that repeated exposure to information at optimal intervals strengthens neural pathways and reduces the rate of forgetting (21).

Cognitive Load Theory provides a complementary lens for understanding why AFC may be particularly effective for certain types of learning tasks (22). This theory distinguishes between intrinsic load (inherent complexity of the material), extraneous load (ineffective instructional design), and germane load (effort devoted to schema construction). AFC’s design—presenting information in discrete, manageable units (flashcards) with immediate feedback—potentially reduces extraneous cognitive load while promoting germane processing through active recall (22, 23). However, the cloze-deletion format commonly used in AFC may inadvertently fragment complex concepts, increasing intrinsic load for integrative understanding (23).

These frameworks inform our hypotheses regarding AFC’s differential effectiveness across exam formats. Specifically, we hypothesize that AFC’s strengths in reducing cognitive load for factual recall tasks may translate to better performance on MCQ-based assessments, while its potential to fragment conceptual understanding may limit its utility for SAQs and OSPEs that require integrative reasoning.

## Materials and methods

This study was designed as a cross-sectional study and conducted at the College of Medicine, Alfaisal University in Riyadh, Saudi Arabia, from September 2023 until January 2024. Convenience sampling was employed to recruit students from 1st to 6th-year medical school during the academic year 2023–2024. The target population comprised all enrolled medical students at Alfaisal University College of Medicine. The required minimum sample size was calculated at 302 participants, based on a 95% confidence level and a 5% margin of error using the online sample size calculator (24, 25). A total of 306 participants were recruited, exceeding the required minimum. Students were recruited through a combination of email invitations sent to official university email addresses and broadcast messages via the student groups on WhatsApp platform for medical students. Invitations were sent on multiple separate occasions to maximize participation. A total of 306 students completed the survey out of approximately 1400 eligible medical students, yielding a response rate of 21.8%. Inclusion criteria included current enrollment in any of the 6 years (Year 1 to Year 6) of the medical program at Alfaisal University College of Medicine. Exclusion criteria included students from other colleges within Alfaisal University (e.g., Engineering, Business, Pharmacy) and medical students who declined to provide informed consent.

A self-administered questionnaire comprising two main sections was distributed among Alfaisal medical students via email and Whatsapp. Participants were asked about their awareness of AFC, frequency and duration of use, specific features found to be most beneficial, and overall satisfaction. The survey also aimed to measure the correlation between AFC usage and GPA levels, ensuring complete anonymity of all participants. The questionnaire was developed in English after reviewing previously published research on the topic (7, 16, 26, 27). Content validity was assessed by having three faculty members with expertise in medical education review each item for clarity, relevance, and coverage of the domain. Face validity was subsequently evaluated through a pilot administration to a convenience sample of 15 medical students who were then excluded from the final study; pilot participants were asked to rate each question for clarity and flag any ambiguous items. Questions were revised based on their feedback before final distribution. Internal consistency of multi-item subscales was assessed using Cronbach’s alpha (28). Cronbach’s alpha for the multi-item subscales (advantages and disadvantages sections) was 0.82, indicating good internal consistency.

Non-Anki users were asked only about their awareness of AFC, reasons for not using it, and their primary study methods. Questions about satisfaction, frequency, duration, and specific features were only presented to self-identified AFC users via skip logic. Inclusion of non-users allowed for comparison of study method preferences and GPA distribution between users and non-users, as well as identification of barriers to adoption.

The questionnaire was voluntary, confidential, and anonymous, allowing only the principal investigator (PI) and co-PI access to responses. Participants were asked to indicate their gender and level of education. Gender data were collected to assess potential differences in AFC usage patterns or perceptions between male and female medical students. The survey consisted a total of 20 closed-ended multiple choice questions, and participants were divided into two groups: AFC users and non-AFC users. A total of 306 students participated in the survey. Ethical approval was obtained from the Alfaisal University’s Institutional Review Board (IRB Approval No. 20248; approved October 2, 2023).

A cross-sectional questionnaire design was chosen to efficiently capture self-reported usage patterns, perceived benefits, and barriers from a large representative sample. While objective usage data from Anki’s platform would be valuable, the questionnaire format allowed for rapid data collection across all 6 years of medical school and assessment of subjective perceptions that are not captured by usage logs alone. We acknowledge that the Anki platform itself provides objective usage statistics (e.g., number of cards reviewed, retention rate, streak length), which were not captured in this study. This limitation is acknowledged in the Limitations section.

Quantitative data (including categorical variables such as gender, year of study, subject areas, and preferred features; and continuous variables such as number of new/review cards per day and CGPA) from the survey was analyzed using the SPSS data analysis package. One-way ANOVA testing was performed to determine statistically significant associations between the academic year of study and AFC usage (29). An independent samples *t*-test was used to identify significant relationships between the mean usage of AFC and variables such as year of study and gender (30). *P*-values < 0.05 were considered statistically significant (31). For the primary analysis examining the relationship between AFC use and academic performance, CGPA was treated as a continuous variable in a multivariable linear regression model, adjusting for potential confounders including gender and year of study. Standardized coefficients (β), 95% confidence intervals (CI), and partial η^2^ are reported as measures of effect size (32, 33).

It should be noted that students who reported using both AFC and traditional study methods were classified as AFC users; the potential confounding effect of concurrent study methods is acknowledged as a study limitation.

## Results

A total of 306 students participated in the survey. Our first set of survey questions assessed the demographics of our respondents, as shown in Table 1. Of the 306 respondents, only 19 did not know about AFC, while the remaining 287 were aware of the app. The demographic characteristics of the participants are summarized in Table 1.

**TABLE 1:** Demographics data of the participants.

Variables	*N* (%)
Gender	
Female	132 (43.1%)
Male	174 (56.9%)
Year of study	
First year	95 (31%)
Second year	58 (19%)
Third year	93 (30.4%)
Fourth year	36 (11.8%)
Fifth year	16 (5.2%)
Sixth year	8 (2.6%)
Users	
AFC users	156 (51%)
Non-AFC users	150 (49%)

**FIGURE 1:**
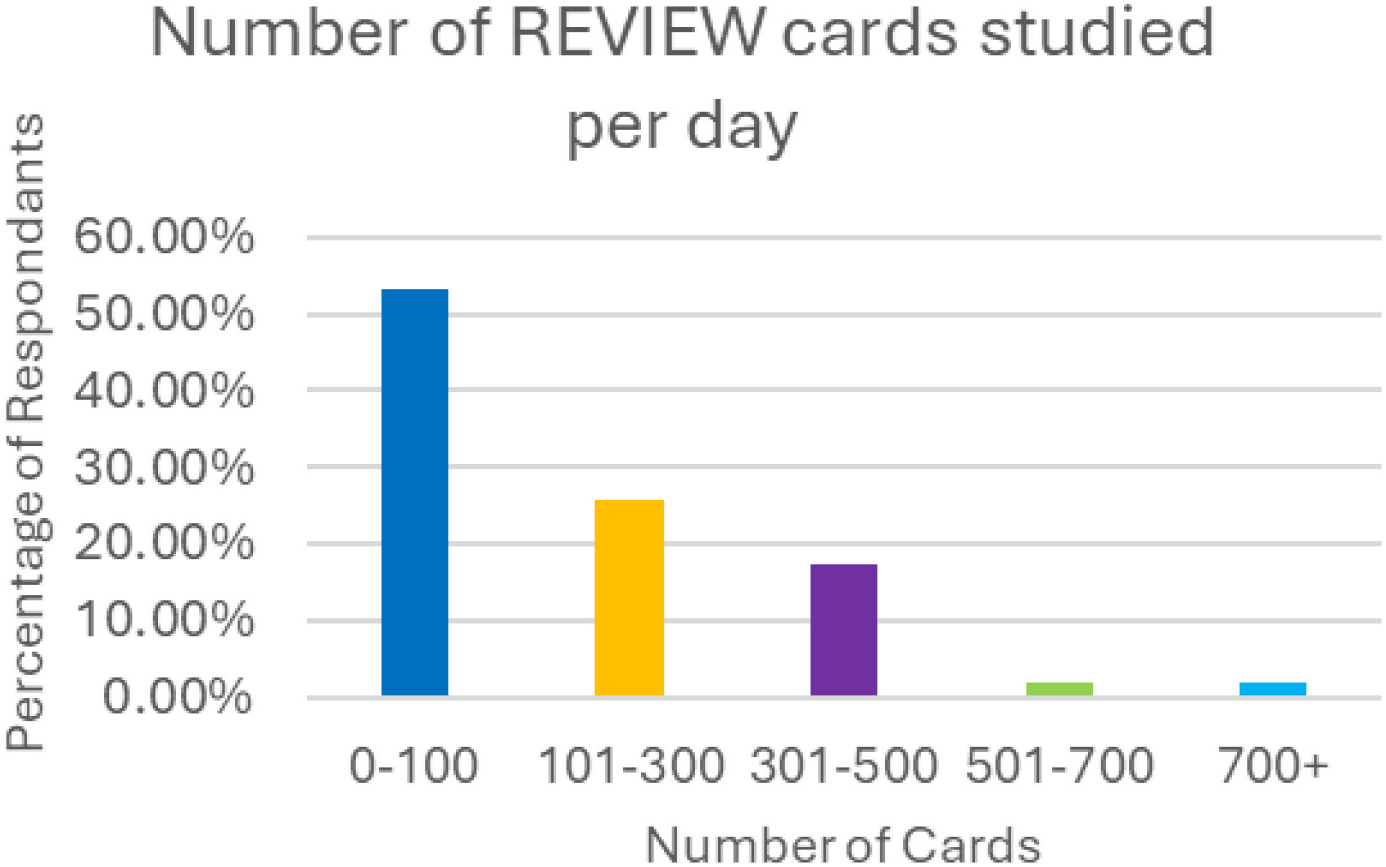
A chart illustrating the number of reviews card studied per day by AFC users participants.

**FIGURE 2:**
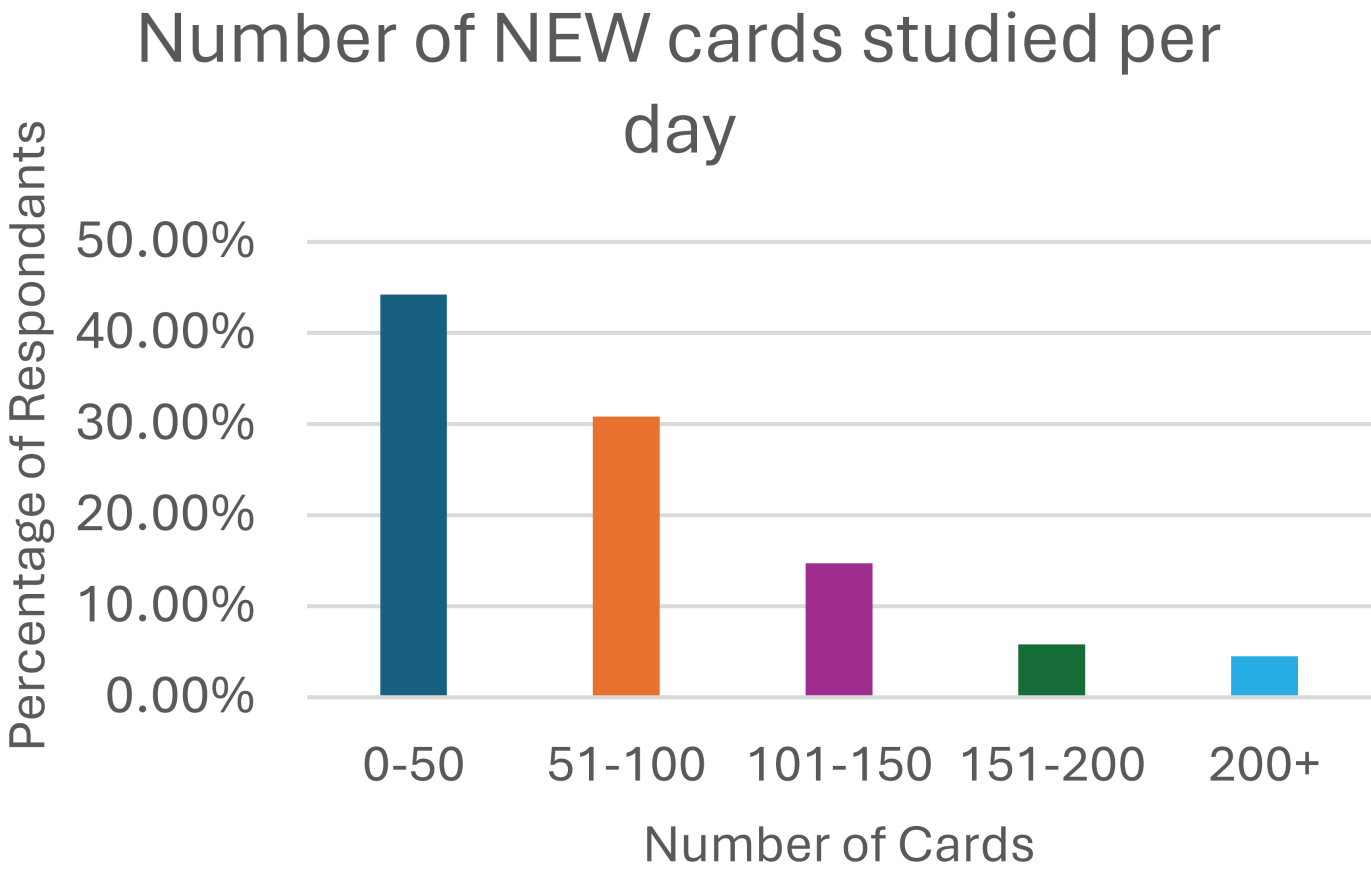
A chart illustrating the number of new cards studied per day by AFC users participants.

We asked users about their frequency of AFC app usage. We inquired, “On average, how many new cards do you study per day?” and “On average, how many review cards do you review per day?” The number of review cards studied per day is shown in Figure 1, and the number of new cards studied per day is shown in Figure 2. The majority of responses indicated studying 1–50 new cards and reviewing 1–100 cards per day. *Post-hoc* correlation analysis revealed no statistically significant correlation between the number of new cards studied per day and CGPA (Spearman’s rho = 0.06, *p* = 0.51, 95% CI: −0.11 to 0.23), nor between review cards per day and CGPA (Spearman’s rho = 0.09, *p* = 0.32, 95% CI: −0.08 to 0.26).

Furthermore, we surveyed students on when they used the AFC app. Our options were during the entire academic year, exams, vacations, and lecture-dense weeks. Our results showed an increase in use during exams, as 55.8% of our population used it during this period compared to 39.7% of users who used it year-round. There was a minimal increase in usage during lecture-dense weeks, as 42.3% of our AFC-using respondents selected this option. There was a significant decrease in the use of AFC during vacations such as the summer break, since only 14.7% of our population reported using the app consistently during this period. Additionally, 73 (46.8%) of users agreed that using AFC decreased their study time.

A total of 117 (75%) of the respondents reported using their colleagues’ AFC cards as a source of study, while 63 (40.4%) of them relied on Anking – a well-known pre-made deck among medical students optimized for USMLE STEPS material – to source their cards. Additionally, 56 (35.9%) of AFC users reported making their own cards too, while only 17 (10.9%) selected the option “Others.”

We further investigated the reasons behind using AFC and its perceived advantages as a study tool. Table 2 illustrates that Anatomy was the most studied subject using AFC (67.3%), followed by Pharmacology (60.9%), Histology (59.0%), and Microbiology (59.0%), highlighting the preference for AFC in basic science disciplines. Table 3 shows that the majority of users (139 out of 156, or 89.1%) selected “Retention of Information” as the primary benefit. Table 4 displays the perceived disadvantages of using AFC. The most chosen option was “Requires Commitment,” with 115 (73.7%) users selecting it. A comparison of preferred study methods between AFC users and non-users is presented in Table 5.

**TABLE 2:** The most studied subjects using AFC Flashcard app.

Subject	Frequency	Percent
Anatomy	105	67.3 %
Physiology	76	48.7 %
Histology	92	59.0 %
Biochemistry	71	45.5 %
Clinical	58	37.2 %
Pharmacology	95	60.9 %
Pathology	76	48.7 %
Microbiology	92	59.0 %
Radiology	39	25.0 %
Embryology	54	34.6 %
Internal Medicine	27	17.3 %
Pediatrics	29	18.6 %
Surgery	23	14.7 %
Obstetrics and Gynecology	25	16.0 %
Others	21	13.5 %

**TABLE 3:** Response distribution on the statement: “I believe the AFC flashcard program has the following advantages” (ONLY USERS).

		Gender			Phase of study		
Advantages	% of Students answered	Male	Female	*P*-value	Preclinical^†^	Clinical^‡^	*P*-value
Retention of information	139 (89.1%)	82 (88.2%)	57 (90.2%)	0.650	108 (90.8%)	31 (83.8%)	0.235
Understanding concepts	46 (29.5%)	27 (29.0%)	19 (30.2%)	0.880	37 (31.1%)	9 (24.3%)	0.430
Customization of the flashcards	54 (34.6%)	28 (30.1%)	26 (41.3%)	0.150	43 (36.1%)	11 (29.7%)	0.474
Track progress	91 (58.3%)	56 (60.2%)	35 (55.6%)	0.562	72 (60.5%)	19 (51.4%)	0.324
Efficiency of use	89 (57.1%)	51 (54.8%)	38 (60.3%)	0.498	68 (57.1%)	21 (56.8%)	0.967
Variety of features	71 (45.5%)	40 (43.0%)	31 (49.2%)	0.446	60 (50.4%)	11 (29.7%)	0.027

† Preclinical: year 1–3 and ^‡^Clinical: year 4–6. All *p*-values were > 0.05, indicating no statistically significant differences between groups, with the exception of ‘Variety of Features’ where preclinical students endorsed this advantage more frequently than clinical students (*p* = 0.027).

**TABLE 4:** Response distribution on the statement: “I believe the AFC flashcard program has the following disadvantages” (ONLY USERS).

			Gender		Phase of study		
Disadvantages	% of Students answered	Male	Female	*P*-value	Preclinical^†^	Clinical^‡^	*P*-value
Requires commitment	115 (73.7%)	70 (75.3%)	45 (71.45%)	0.593	91 (76.5%)	24 (64.9%)	0.161
Difficult to understand	30 (19.2%)	22 (23.7%)	8 (12.7%)	0.088	21 (17.6%)	9 (24.3%)	0.363
Advanced settings	46 (29.5%)	26 (28%)	20 (31.7%)	0.611	39 (32.8%)	7 (18.9%)	0.106
Errors in cards	38 (24.4%)	20 (21.5%)	18 (28.6%)	0.313	27 (22.7%)	11 (29.7%)	0.384
Time-consuming	79 (50.6%)	46 (49.5%)	33 (52.4%)	0.721	57 (47.9%)	20 (54.1%)	0.513

† Preclinical: year 1–3 and ^‡^Clinical: year 4–6. All *p*-values were > 0.05, indicating no statistically significant differences between groups.

**TABLE 5:** Perceived correlation between AFC use and academic performance.

	AFC users		Non-AFC users	
Methods	Frequency	Percent	Frequency	Percent
Studying lecture slides	104	66.7	103	78.6
Note taking and summaries	69	44.2	75	57.3
Practice questions	128	82.1	98	74.8
External resources	103	66.0	59	45
Others	9	5.8	7	5.3

To compare AFC to other study methods, we asked 287 students who use AFC about their preferred methods. Among them, 144 selected “Note Taking and Summaries,” 207 selected “Lecture Slides,” 228 selected “Practice Questions,” 162 selected “External Resources,” and 16 selected “Others.” The “Others” category included responses such as group study sessions, video-based learning (e.g., YouTube, Osmosis), and textbook reading.

To assess if students perceived a correlation between AFC use and academic performance, we surveyed them using a 5-point Likert scale asking if they noticed any correlation. Of the respondents, 58 (37.2%) selected “Agree” and 43 (27.6%) selected “Strongly Agree,” while 51 (32.7%) participants selected “Neutral.” Only 4 participants selected “Disagree,” and “Strongly Disagree” was not chosen by any.

### Multivariable regression analysis of CGPA

To rigorously examine the relationship between AFC use and academic performance, we conducted a multivariable linear regression analysis with CGPA as a continuous dependent variable. The independent variables included AFC user status (yes/no), gender, and year of study. The model was statistically significant [*F*(3, 210) = 4.21, *p* = 0.006, adjusted R^2^ = 0.044], indicating that the included variables collectively explained approximately 4.4% of the variance in CGPA.

The regression coefficients are presented in Table 6. After adjusting for gender and year of study, AFC use was not significantly associated with CGPA (β = 0.04, 95% CI: −0.08 to 0.16, *p* = 0.52, partial η^2^ = 0.002). Gender was significantly associated with CGPA, with female students having higher CGPA on average compared to male students (β = 0.21, 95% CI: 0.06 to 0.36, *p* = 0.006, partial η^2^ = 0.034). Year of study was also significantly associated with CGPA (β = −0.18, 95% CI: −0.30 to −0.06, *p* = 0.004, partial η^2^ = 0.038), indicating a trend of declining CGPA in later years of medical school.

**TABLE 6:** Multivariable linear regression analysis of factors associated with CGPA.

Variable	β (Standardized)	95% Confidence interval	*p*-value	Partial η^2^
AFC user (vs. non-user)	0.04	−0.08 to 0.16	0.52	0.002
Gender (female vs. male)	0.21	0.06 to 0.36	0.006	0.034
Year of study	−0.18	−0.30 to −0.06	0.004	0.038

Model summary: *F*(3, 210) = 4.21, *p* = 0.006, Adjusted R^2^ = 0.044.

We further measured the correlation between AFC usage and having a higher GPA. Of the 306 participants, 214 (69.9%) agreed to disclose their CGPA. CGPA (Cumulative Grade Point Average) reflects performance across all semesters of medical school. We acknowledge that a single-semester intervention or variable usage patterns may not sufficiently influence cumulative GPA, which aggregates performance over multiple years. This is an important limitation, and future studies should examine course-specific grades in relation to AFC use for that particular course. Among those who reported their GPA, 92 students had a CGPA below 3.5. Of these, 51 (55.4%) were AFC users, and the remaining 41 (44.6%) were not.

Similarly, 56 students reported a CGPA between the 3.5−3.75 range. Of these, 33 (58.9%) were AFC users, while the remaining 23 (41.1%) were not. Finally, 66 students reported a CGPA between 3.75−4.0. Among them, 39 (59.1%) were AFC users, while the remaining 27 (40.9%) were not. When comparing mean CGPA between groups, AFC users had a mean CGPA of 3.59 (SD = 0.36), while non-users had a mean CGPA of 3.55 (SD = 0.39); this difference was not statistically significant [t(212) = 0.76, *p* = 0.45, Cohen’s d = 0.10, 95% CI for difference: −0.08 to 0.16].

## Discussion

AFC relies heavily on spaced repetition, a learning strategy focused on optimizing long-term memory retention. This technique involves the systematic reiteration of information at progressively increasing intervals, dictated by an algorithm (18, 34). This approach contrasts with cramming and leverages the spacing effect to enhance knowledge consolidation. AFC utilizes this concept by creating a system for presenting cards at the most suitable time. This system divides cards into learning and graduated categories. Learning cards reappear at fixed time intervals. After a certain number of fixed intervals, a card becomes graduated, meaning it appears with varying time intervals based on an algorithm within the Anki software. This algorithm and the time interval for card reappearance are affected by the user’s response. For instance, if the user selects “good,” the time interval for the card’s reappearance increases, and vice versa. Research on spaced repetition broadly supports the effectiveness of this approach for long-term information retention (18, 34); however, the software itself can be challenging to learn and get accustomed to. This can lead to a significant time investment for students to transition to this method, especially when they have limited time to experiment with new study techniques (18).

### GPA

Our results showed that although students who used AFC perceived a connection to higher academic performance, we found no statistically significant correlation (*P*-Value > 0.05) between higher cumulative GPAs and increased AFC usage. This null finding was confirmed by our multivariable regression analysis, which treated CGPA as a continuous variable and adjusted for potential confounders (gender and year of study). The standardized coefficient for AFC use was β = 0.04 (95% CI: −0.08 to 0.16, *p* = 0.52, partial η^2^ = 0.002), indicating that AFC use explained less than 1% of the variance in CGPA after accounting for other factors. The effect size (Cohen’s d = 0.10) was negligible, further supporting the absence of a meaningful association. We recognize that reliance on self-reported perception data and CGPA is a significant limitation. The absence of an objective, prospective, course-specific measure of academic performance tempers the conclusions that can be drawn. The discussion emphasizes that our primary contribution is describing usage patterns and perceived benefits rather than establishing causal effectiveness. It is essential to emphasize that this is a correlational finding and no causal relationship can be inferred from this cross-sectional study. Students reported using AFC concurrently with their coursework, not prior to it. The CGPA reflects performance on exams taken during the same period in which students reported using AFC. However, the cross-sectional design cannot establish temporal precedence or causation.

This null finding appears to diverge from several prior studies; however, those studies typically examined performance on single, standardized MCQ-format assessments, whereas our outcome measure (CGPA) aggregates performance across multiple exam types including MCQs, SAQs, and OSPEs. This methodological difference likely accounts for the discrepancy. For example, Lu et al. reported a positive correlation between higher USMLE STEP 1 scores and AFC use (7). Similarly, Lambers et al. found a positive correlation between AFC use and passing the orthopedics exam (11). The only study aligning with our results is a recent pilot study by Levy et al., which showed no significant difference in scores between frequent-AFC users and limited-AFC users (35).

These articles have investigated the correlation between AFC use and achieving a higher score on a single test that typically uses a single format, namely MCQs. Unlike these tests (such as STEP 1), our university exams usually consist of three components: MCQs (multiple choice questions), SAQs (Short Answer Questions), and OSPEs (Objective Structured Practical Examinations). Therefore, by examining the correlation of AFC with our students’ GPAs, we are exploring how effective AFC is with different exam styles.

**FIGURE 3:**
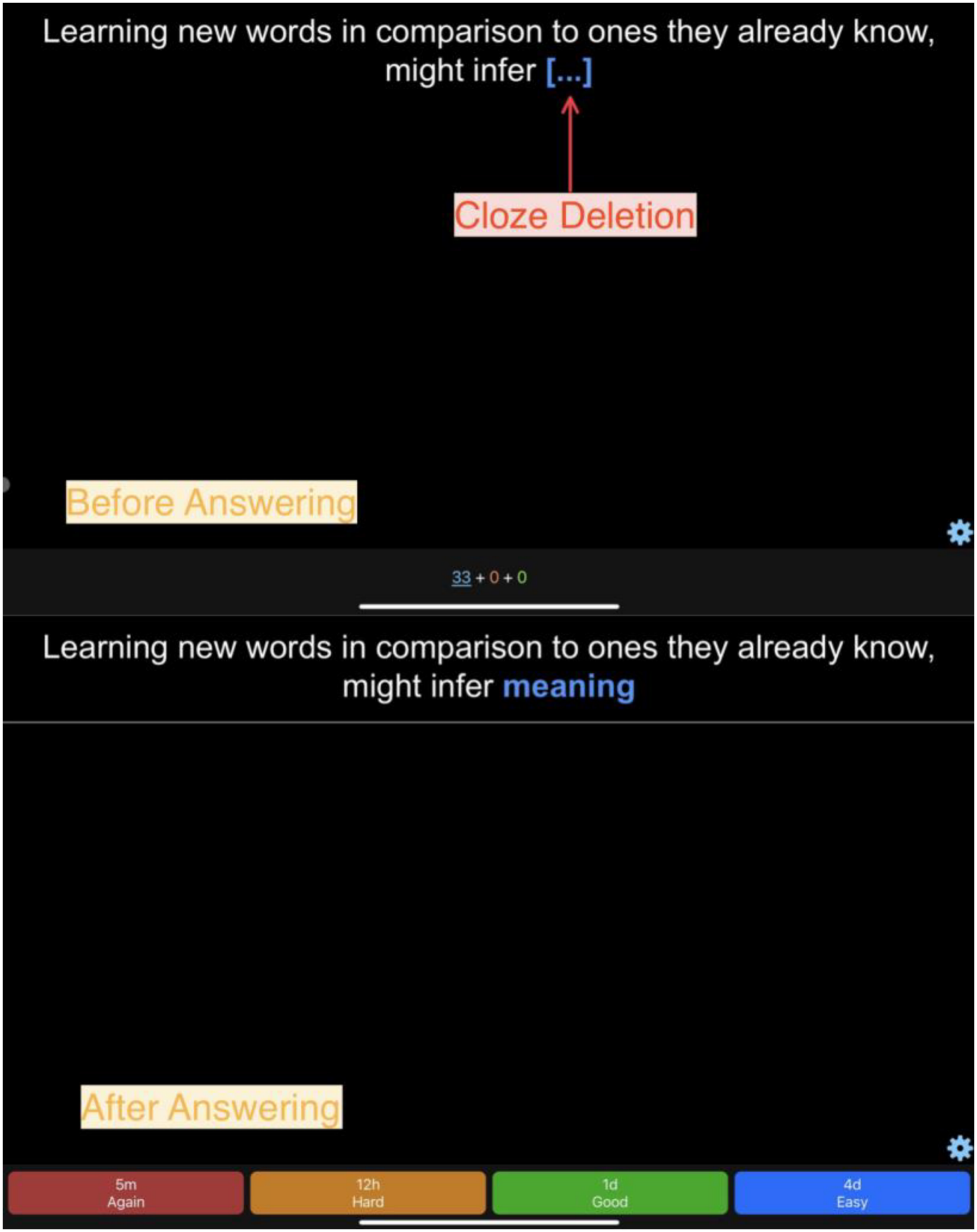
Cloze Deletion card. The ellipsis within the bracket (blank area) are replaced with the phrase upon pressing on the card.

One potential explanation for this pattern relates to the match between AFC’s design and MCQ-based assessment formats. Mee et al. discussed the idea that students can answer mainly by recognizing key terms in the clinical vignette and matching them with the best possible answer (36). The majority of AFC’s flashcards are created in the cloze deletion format, which is technically fill-in-the-blank. Thus, this memorization method aids in identifying key terms in concepts since the clozed parts (the blank parts that the user should fill in) are usually the key terms on the cards. This method is shown in Figure 3. We suspect that many users rely on recognizing the key term from a card rather than learning the entire concept, which serves students well in MCQs.

Conversely, the literature (15, 36) and our own findings suggest AFC may be less suited to short answer questions, as flashcard-based memorization of isolated key terms does not necessarily develop the conceptual understanding required to construct a complete written response. This is supported by participants’ responses: “Understanding Concepts” was the least selected advantage of AFC, with only 26.9% of respondents choosing it. It is important to emphasize, however, that this explanation remains inferential; the study design does not permit causal conclusions, and the observed pattern warrants further investigation in prospective studies that directly measure performance on different exam formats.

Furthermore, many flashcards break down a single concept into various cards to make them easier to retain. This can further exacerbate the problem of understanding entire concepts as a single entity. It’s true that even in SAQs students need to write down the key terms in their answers, but they also need to explain an entire concept, such as the pathophysiology of a disease. A study by Sam et al. demonstrated that answering a VSAQs (Very Short Answer Questions) question requires the ability to “generate a piece of knowledge in the absence of cues.” (37). Answering an SAQ or any written format question may pose a problem for students who rely heavily on AFC for their studies. This hypothesis might explain why there is no correlation with our students’ CGPA, while other articles reported a positive correlation with other exams (7, 9, 11).

The study by Lambers et al. only had 12 participants in their prospective study. This small sample size raises concerns that their results might be skewed (11). While Lu et al. collected more almost 200 responses in their cross-sectional study,132 of their respondents were AFC users, while only 69 were not. This large gap suggests students who are more engaged with AFC might have been more likely to participate in the survey (7). To address this bias, we took steps to ensure equal participation from both groups. We specifically solicited responses from regular AFC users and non-users. This strategy resulted in an even distribution: 150 AFC users and 156 non-users. Furthermore, our study boasts a more diverse population. It included students from all years, from first year to internship. This, along with the larger sample size compared to other studies on the same topic, suggests our findings hold greater accuracy.

The higher proportion of AFC users in preclinical years (Years 1–3) compared to clinical years (Years 4–6) likely reflects the fact that preclinical curricula emphasize foundational basic science knowledge (anatomy, physiology, pharmacology, pathology, microbiology), which is well-suited to flashcard-based memorization. Clinical years require more integrative reasoning, history-taking, physical examination skills, and patient management—domains less amenable to flashcard review. Additionally, clinical students have less structured study time and greater in-hospital responsibilities. This pattern is consistent with Table 2, which shows that basic science subjects (Anatomy, Histology, Pharmacology, Microbiology) were among the most frequently studied using AFC.

### Advantages and disadvantages of AFC

Another important aspect we examined in this study is the advantages and disadvantages of AFC. Jape et al. mentioned in their article that students, although aware of the benefits of spaced repetition (the hallmark of flashcard software like AFC), struggle to incorporate these techniques into their study routines due to time management issues associated with implementing new methods (13). Our findings align with theirs, as respondents most frequently selected “Requires Commitment” and “Time-consuming” when asked about AFC’s disadvantages.

AFC heavily relies on spaced repetition, a learning strategy focused on optimizing long-term memory retention. This technique involves the systematic reiteration of information at progressively increasing intervals, dictated by an algorithm (18). This approach contrasts with cramming and leverages the spacing effect to enhance knowledge consolidation. AFC utilizes this concept by creating a system for presenting cards at the most suitable time. This system divides cards into learning and graduated categories. Learning cards reappear at fixed time intervals. After a certain number of fixed intervals, a card becomes graduated, meaning it appears with varying time intervals based on an algorithm within the AFC software. This algorithm and the time interval for card reappearance are affected by the user’s response. For instance, if the user selects “good,” the time interval for the card’s reappearance increases, and vice versa. Research on spaced repetition broadly supports the effectiveness of this approach for long-term information retention; however, the software itself can be challenging to learn and get accustomed to. This can lead to a significant time investment for students to transition to this method, especially when they have limited time to experiment with new study techniques.

AFC’s effectiveness relies on the user’s commitment to applying spaced repetition. As explained earlier, more exposure to a card leads to longer intervals between reviews. Thus, the AFC software further classifies cards after graduation into young and mature categories. Mature cards have an interval of 21 days or more, meaning they appear every 21 days or more, while young cards have an interval of less than 21 days (34). This classification is designed to help students identify when a card’s information has been sufficiently reinforced for long-term retention. Ideally, all cards should be mature before an exam to ensure the material is well-memorized.

Additionally, AFC is designed for daily use because any skipped days cause cards to accumulate for the next day. For example, if a user skips 300 due cards on Tuesday, they will have the 300 cards from the previous day in addition to the cards scheduled to appear on Wednesday. Ideally, users should use AFC on a daily basis.

These factors make it difficult for students to stay on track with AFC, as it requires a long-term commitment to reap the benefits of the effort invested in the application. Consequently, many students avoid relying on it, fearing the commitment (13). Others get overwhelmed by the number of due cards they have to complete if they skip a day and cards accumulate. It is worth noting that there are add-ons, which are additional pieces of code downloaded separately that integrate with the original software to modify or add certain features, to solve the card accumulation issue. These add-ons are created by independent entities.

Our results showed that 75% of students used colleagues’ cards, which may contain material outside course syllabi. This highlights an opportunity for faculty-supervised, curriculum-aligned flashcard repositories to ensure content relevance.

### Frequency of use

Harris et al. recruited 60 participants at the University of Central Florida (UCF) to study AFC usage among first-year medical students in a Structure and Function course (5). Their results showed that 70% of the participants used AFC weekly, and 30% reviewed more than 800 cards weekly. On the other hand, we reported that 51% of respondents are regular AFC users. Of these regular users, 53.2% review 1–100 cards daily, and 44.2% learn 1–50 new cards per day. Our results suggest that students find the 100–300 review and 50–100 new card range suitable because most students would prefer not to do more cards in a single sitting. This is because reviewing a large number of cards at once, along with studying new cards, can be overwhelming and time-consuming. Additionally, since most students integrate AFC as a supplement to their studies, these averages seem reasonable for that purpose.

### Popularity

Our research investigated the awareness of AFC among medical students at Alfaisal University. We found that only 19 out of 306 surveyed students were not familiar with the application. This shows a very high level of awareness among the students even though a third of the participants were in their first year when the survey was conducted. This widespread awareness is primarily due to how student organizations promote the use of such supplementary materials as one of the student bodies known as Alfaisal University’s Medical Students Association (MSA) has a dedicated AFC team. This team creates and distributes flashcards for courses taught in years one to three, while also offering tutorials on how to use the software. These initiatives encourage students to explore and utilize such tools.

Furthermore, we have observed a growing online trend promoting AFC within medical communities. Dedicated YouTube channels and Reddit forums exist solely for AFC discussions. Additionally, articles like the one published by Hart-Maytag et al. provide valuable guidance on creating and using AFC flashcards (17). All these factors contribute to the increased adoption of AFC among medical students.

## Recommendations for students and educators

Based on our findings, we offer the following recommendations:

For medical students:

1. Use AFC as a supplementary tool rather than a primary learning resource, particularly for memorization-heavy subjects such as anatomy, pharmacology, and microbiology.
2. Prioritize creating or using curriculum-aligned flashcards to avoid extraneous content that may increase cognitive load and test anxiety.
3. Maintain consistent daily usage to prevent card accumulation and optimize spaced repetition benefits.
4. Supplement AFC with active learning strategies such as practice questions and conceptual mapping, especially when preparing for SAQs and OSPEs.

For medical educators:

1. Consider providing faculty-reviewed, course-specific flashcard decks to ensure content accuracy and curricular alignment.
2. Recognize the limitations of flashcards for assessing conceptual understanding and balance exam formats accordingly.
3. Offer workshops or tutorials on evidence-based study techniques, including spaced repetition and active recall, to help students use AFC effectively.
4. Encourage integration of AFC with other study methods rather than reliance on flashcards alone.

### Limitations

Our study has several important limitations that must be acknowledged. First, due to its cross-sectional and observational design, causal inference between AFC usage and academic performance cannot be established; the correlation-causation distinction is central to interpreting these findings. The cross-sectional design does not allow for pre-post comparisons. We did not measure baseline academic performance prior to AFC adoption. Future prospective studies should employ a longitudinal design with baseline and follow-up assessments. Second, the data were collected from a single university via convenience sampling, which limits generalizability to other institutions or educational systems. Third, approximately one-third of respondents declined to disclose their CGPA, introducing possible non-response bias in the GPA analysis. Fourth, the questionnaire relied entirely on self-reported data, including perceived AFC usage frequency and subjective perceptions of academic benefit, which are subject to recall bias and social desirability effects. The questionnaire assessed self-reported frequency and patterns of use. We acknowledge that the AFC platform itself provides objective usage statistics (e.g., number of cards reviewed, retention rate, streak length), which were not captured in this study. Future research should integrate these objective metrics. Fifth, students who used AFC concurrently with other study methods were classified as AFC users, making it difficult to isolate the independent effect of AFC. Future studies should attempt to separate exclusive AFC users from mixed-method users. Sixth, our university’s exams include MCQs, SAQs, and OSPEs, making GPA a heterogeneous outcome; the analysis was not stratified by exam format, which may have diluted any MCQ-specific effect of AFC. We did not examine course-specific or exam-specific performance. CGPA serves as a global measure that may dilute subject-specific effects. Future studies should correlate AFC use with performance in individual courses or specific exam formats (e.g., MCQ-only examinations). Seventh, while we applied multivariable regression, the relatively small sample size for the regression analysis (*n* = 214 for those who disclosed CGPA) may have limited statistical power to detect small effect sizes. The observed effect size for AFC use (β = 0.04) was very small, and the confidence interval included zero, indicating that even with greater statistical power, a meaningful association is unlikely. Finally, the study did not objectively measure knowledge retention, and future research incorporating objective testing alongside self-report data would provide more robust conclusions about AFC’s educational value.

## Conclusion

This cross-sectional study found no statistically significant correlation between AFC use and CGPA among medical students at Alfaisal University, though a majority of users perceived subjective academic benefits. Given the observational design, causal conclusions cannot be drawn. Students appear to use AFC predominantly for memorization-based and basic science subjects, likely due to the availability of pre-made decks for these areas. AFC may function most effectively as a supplementary tool for MCQ-heavy assessments rather than as a universal academic performance enhancer. Future research should employ prospective longitudinal designs, objectively measure knowledge retention, stratify outcomes by exam format, and investigate AFC’s impact in diverse, non-US medical education contexts to build a stronger evidence base for its integration into medical curricula.

## Data Availability

The original contributions presented in this study are included in this article/supplementary material, further inquiries can be directed to the corresponding author.
